# Chromosome-scale assembly of the African yam bean genome

**DOI:** 10.1038/s41597-024-04210-2

**Published:** 2024-12-18

**Authors:** Bernice Waweru, Isaac Njaci, Rajneesh Paliwal, Mary Maranga, Collins Muli, Edwin Murungi, Davies Kaimenyi, Beatus Lyimo, Helen Nigussie, Bwihangane Birindwa Ahadi, Ermias Assefa, Hassan Ishag, Oluwaseyi Olomitutu, Michael Abberton, Christopher Darby, Cristobal Uauy, Nasser Yao, Daniel Adewale, Peter Emmrich, Jean-Baka Domelevo Entfellner, Oluwaseyi Shorinola

**Affiliations:** 1 International Livestock Research Institute, P.O. Box 30709, Nairobi, 00100 Kenya; 2 John Innes Centre, Norwich Research Park, Norwich, NR4 7UH UK; 3 School of Agriculture and Food Sustainability, The University of Queensland, Brisbane, QLD Australia; 4 Genetic Resources Center, International Institute of Tropical Agriculture, Oyo Road, Ibadan, 200001 Nigeria; 5 Department of Biochemistry, Jomo Kenyatta University of Agriculture and Technology, P.O. Box 62000, Nairobi, 00200 Kenya; 6 Malopolska Centre of Biotechnology, Jagiellonian University, Krakow, Poland; 7 Department of Medical Biochemistry, Kisii University, P.O. Box 408-40200, Kisii, Kenya; 8 Bioscience Research Centre (PUBReC), Pwani University, P.O Box 195-80108, Kilifi, Kenya; 9 Institut für Mikrobiologie und Biochemie, Hochschule Geisenheim University, Von-Lade-Str. 1, 65366 Geisenheim, Germany; 10 Nelson Mandela African Institute of Science and Technology, Arusha, Tanzania; 11 Department of Microbial Cellular and Molecular Biology, Addis Ababa University, Addis Ababa, Ethiopia; 12 Université Evangélique en Afrique, UEA, Faculty of Agriculture and Environment sciences, Bukavu, Democratic Republic of the Congo; 13 Université Officielle de Bukavu, UOB, Faculty of Sciences, Bukavu, Democratic Republic of the Congo; 14 Department of Agronomy, Horticulture and Plant Science, South Dakota State University, Brookings, USA; 15 College of Veterinary Sciences, University of Nyala, Nyala, Sudan; 16 Biosciences eastern and central Africa-International Livestock Research Institute (BecA-ILRI) Hub, International Livestock Research Institute, Nairobi, Kenya; 17 Department of Crop Science and Horticulture, Federal University Oye-Ekiti, Ikole-Ekiti Campus, Nigeria; 18 Norwich Institute for Sustainable Development, School of Global Development, University of East Anglia, Norwich, NR4 7TJ UK; 19 School of Bioscience, University of Birmingham, Edgbaston, Birmingham B15 2TT UK

**Keywords:** Plant breeding, Conservation genomics, Agriculture

## Abstract

Genomics-informed breeding of locally adapted, nutritious, albeit underutilised African crops can help mitigate food and nutrition insecurity challenges in Africa, particularly against the backdrop of climate change. However, utilisation of modern genome-assisted crop improvement tools including genomic selection and genome editing for many African indigenous crops is hampered by the scarcity of genomic resources. Here we report on the assembly of the genome of African yam bean (*Sphenostylis stenocarpa)*, a tuberous legume crop that is indigenous to Africa. By combining Nanopore-based assembly with Hi-C scaffolding, we produced a high-quality chromosome-scale assembly with an N50 of 69.5 Mbp. Using transcriptome evidence from Nanopore RNASeq and protein homology evidence from related crops, we predicted and annotated 31,614 putative protein coding genes. We also show how this genome substantially improves anchoring of genetic markers from African yam bean, confirming its significance as a resource for genetic research in African yam bean.

## Background and Summary

**Fig. 1:**
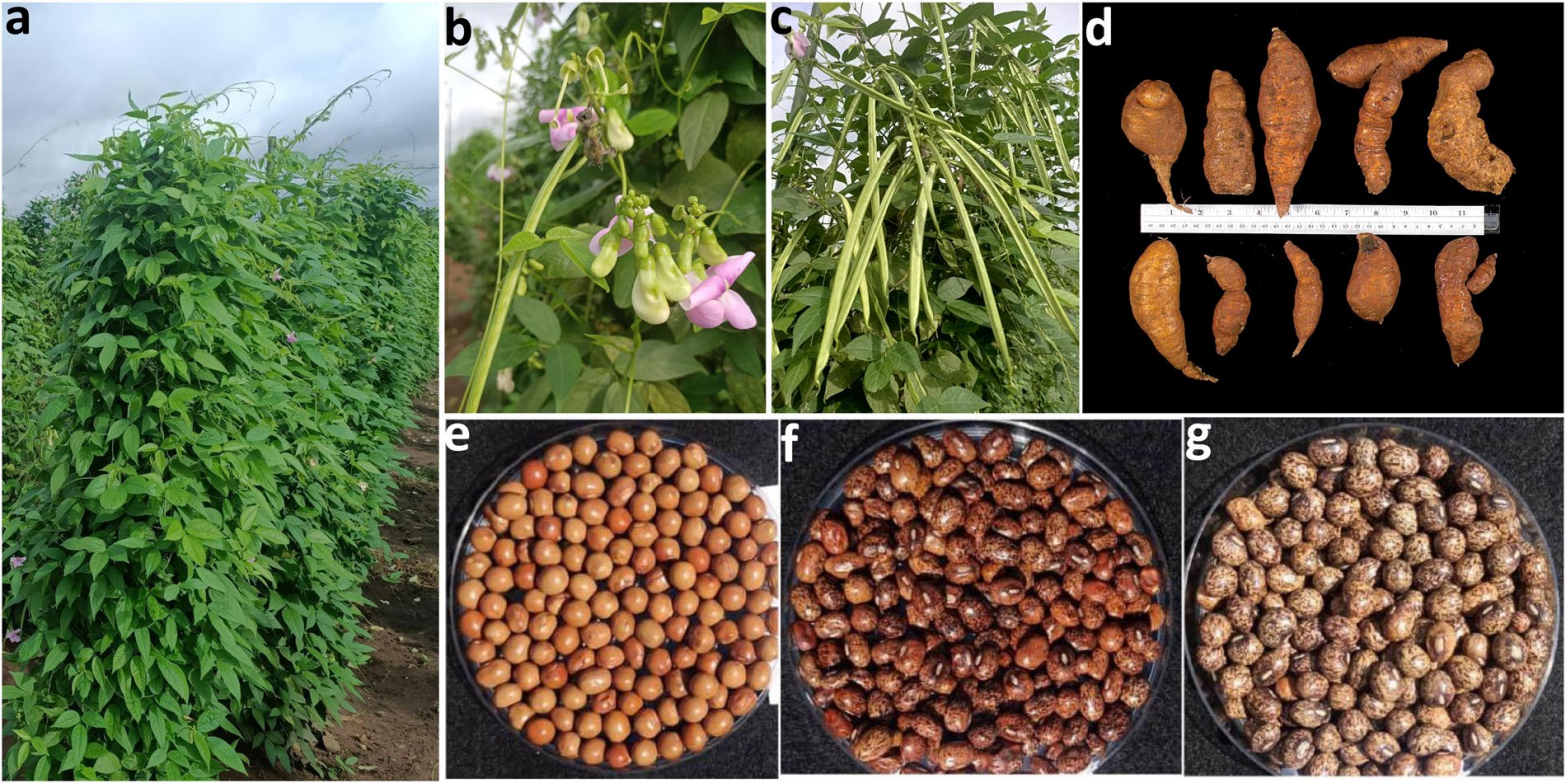
Africa yam bean - an African indigenous tuberous legume. Figure shows (**a**) full grown plants in the field (**b**) flowers (**c**) pods (**d**) root tubers of different shapes and sizes, (**e**–**g**) different coloured seeds.

African yam bean (*Sphenostylis stenocarpa* (Hochst. Ex. A. Rich) Harms) is an underutilised tuberous legume, which produces edible protein-rich seeds and starch-rich tubers (Fig. 1). It is a tropical African crop^1^ that originated from Ethiopia from where its distribution extended to West and Central Africa^2^. African yam bean (hereafter referred to as AYB) is important for food and nutritional security in local communities in sub-saharan Africa. AYB is a self-pollinating diploid crop^3,4^ (2n = 22) that is a rich source of dietary protein with up to 30% and 10% protein content in the seeds and tubers, respectively. Its seeds and tubers are also low in fat and rich in carbohydrates, minerals and vitamins^5,6^. In addition, AYB exhibits high nitrogen-fixing ability^7^ and is drought tolerant. These attributes may have allowed it to thrive in marginal soils under low-input farming systems and intercropping, especially in Ghana and Nigeria^8,9^.

AYB, however, is largely underutilised due to the hardness of the seed coat, leading to long cooking time and the presence of anti-nutritional factors, which reduce protein digestibility^5^. Also, the need for staking of plants has greatly hampered its cultivation on a commercial scale. Its production has been sustained indigenously through intercropping with major crops, especially yam - *Dioscorea spp*. To date, minimal genomic information is available to assist breeding efforts aimed at unlocking the full potential of AYB, thereby limiting its contribution to food and nutritional security in Africa.

**Fig. 2:**
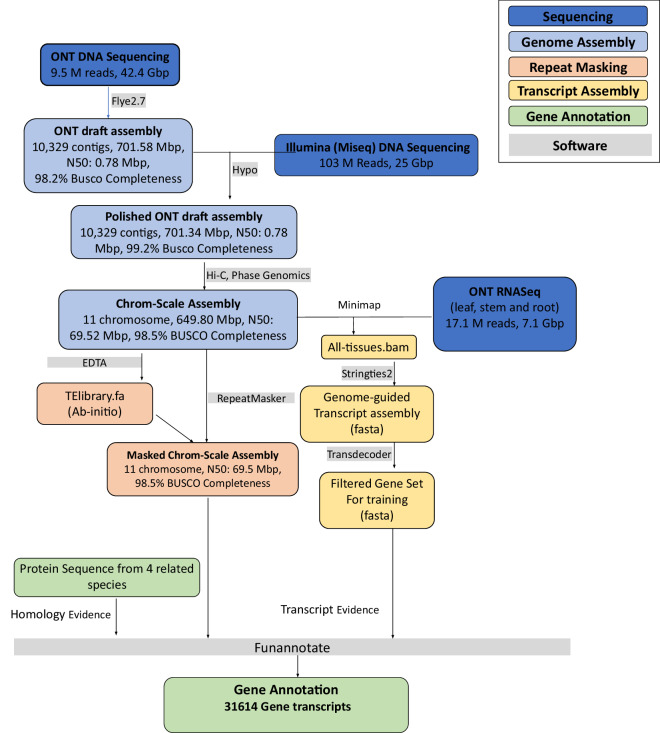
AYB genome sequencing and annotation workflow. Overview of the workflow used for the sequencing, assembly, masking and annotation of the African Yam Bean genome. The boxes are color-coded by the different stages involved in the workflow (top right box). Software used for each step are indicated in grey boxes.

Here, we present a chromosome-scale assembly of the AYB genome primarily using Oxford Nanopore long reads and scaffolded using high-throughput chromosome conformation capture (Hi-C) scaffolding (Fig. 2). Using homology and transcript evidence, we performed gene annotation of the AYB genome (Fig. 2). We further demonstrate the usefulness of this genome resource for genetic analyses in AYB.

## Methods

### Size estimation of AYB genome by flow cytometry and kmer analyses

**Fig. 3:**
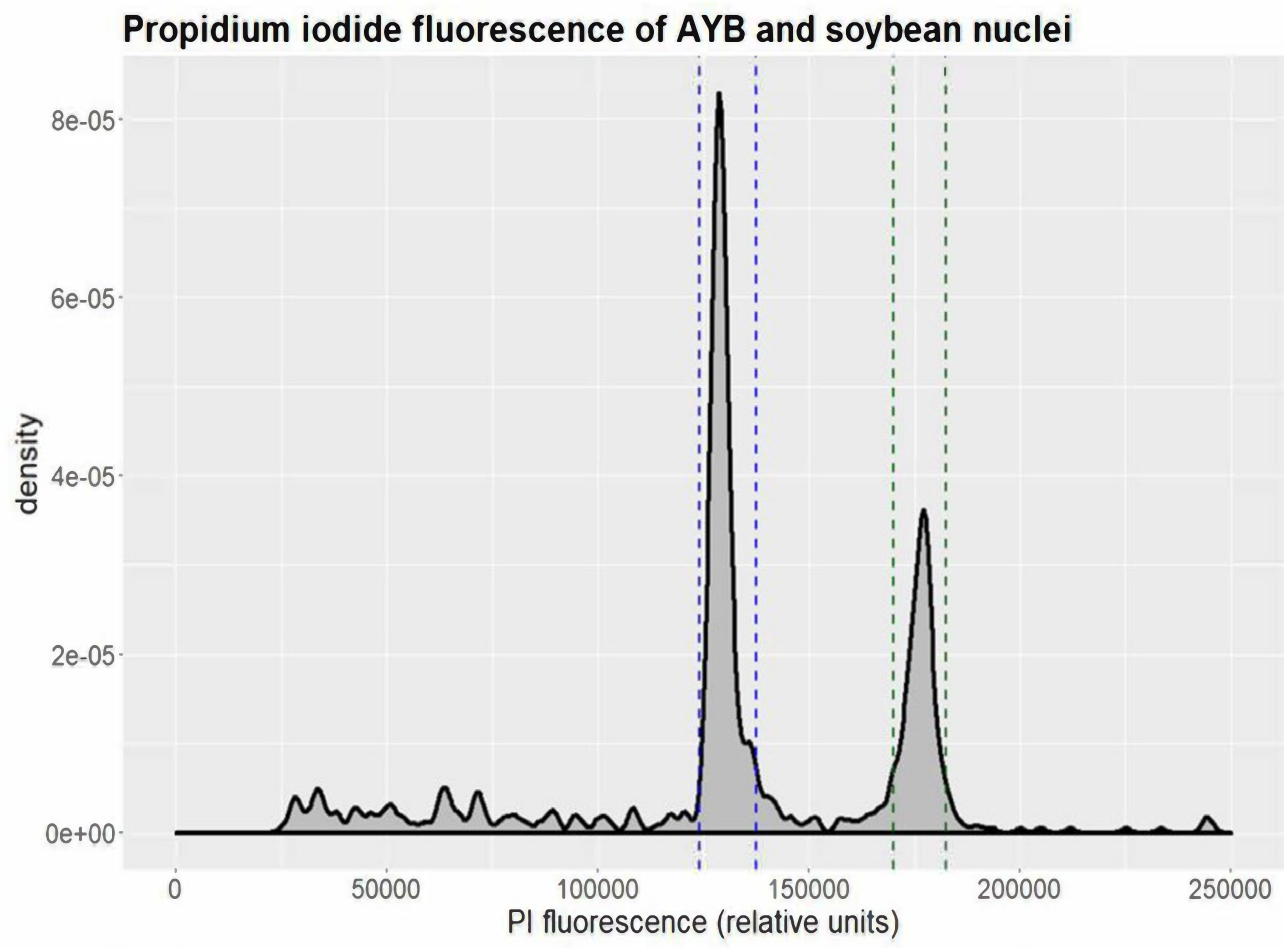
Genome size estimation of AYB. Density plot showing results of a representative flow cytometry run, excluding events caused by cell debris. Propidium iodide fluorescence amplitude (in relative units) is plotted against event density. The interval assumed to be AYB nuclei is delimited by blue dashed lines, the interval assumed to be soybean nuclei is delimited by green dashed lines. Three biological replicates were performed.

We used both flow cytometry and kmer analyses to estimate the genome size of AYB. For the flow cytometry analyses, fresh 10 mg leaf samples of AYB (accession TSs11^10^) and soybean (*Glycine max*, used as standard) were immersed in 1 mL of ice-chilled Galbraith buffer (45 mM MgCl_2_, 30 mM sodium citrate, 20 mM 3-(N-morpholino) propanesulfonic acid, 0.1% w/v Triton X-100, pH 7) and sliced using a scalpel. The supernatant was filtered through one layer of Miracloth (pore size 22 - 25 µm). An aliquot of 600 µL of filtrate was mixed with propidium iodide to a concentration of 50 µM and RNAse A to 20 µg/mL and incubated for 1.5 h on ice. A FACSCantoll flow cytometer (Becton Dickinson) was used to measure nuclei, with flow rate adjusted to between 20 and 50 events/s, and results were analysed using FCSalyser (v. 0.9.18 alpha). The genome size of AYB was estimated following the method described by Dolezel *et al*.^11^ by dividing the mean position of its fluorescence peak by the mean position of the corresponding soybean peak, and multiplying by the estimated soybean genome size of 1.10–1.15 Gbp^12^ (Fig. 3). Based on this range we estimate the size of the AYB genome as 804–841 Mbp. This analysis was performed with three biological replicates.

For kmer-based genome size estimation, the distribution of 20-mer was calculated using Jellyfish based on Illumina sequencing data from the same accession (described below). The total number of kmers obtained from the sequencing data was 15,257,864,252. After removing kmers with abnormal depth (likely from sequencing errors), 15,038,011,105 kmers were used for genome size estimation, with the main peak in kmer depth at 21. The genome size was inferred by dividing the total number of kmers by the kmer depth^13^. The estimated genome size of AYB was ~716 Mbp. The discrepancies between the genome size estimates from flow cytometry and kmer analysis could be due to the uncertainty of the genome size of the non-reference soybean cultivar used as standard in the flow cytometry estimation. Variation in genome size has been reported in Soybean with modern cultivars shown to have less gene content than old cultivars^14^. Importantly, the k-mer analysis provides an intrinsic genome size estimate directly derived from AYB sequence data.

### Sample selection, library preparation and sequencing

#### Nanopore DNA sequencing

Two grams of young leaves of a single plant of AYB accession TSs11 were harvested, frozen in liquid nitrogen and stored at −80 °C. The leaves were then ground in liquid nitrogen using a pestle and mortar. High Molecular Weight (HMW) DNA was extracted from the ground sample with Carlson lysis buffer (100 mM Tris-HCl, pH 9.5, 2% CTAB, 1.4 M NaCl, 1% PEG 8000, 20 mM EDTA) followed by purification using the Qiagen Genomic-tip 100/G as described in the Oxford Nanopore Technologies (ONT, UK) HMW plant DNA extraction protocol^15^. The ONT SQK-LSK109 ligation sequencing kit protocol was used to prepare sequencing libraries from the HMW DNA. For each run, this involved repairing and 3’ adenylation of 1 µg of HMW genomic DNA with the NEBNext FFPE DNA Repair Mix and the NEBNext® Ultra™ II End Repair/dA-Tailing Modules (New England Biolabs, NEB, UK). Sequencing adapters were then ligated using the NEBNext Quick Ligation Module (NEB, UK). After library purification with AMPure XP beads (Beckman Coulter), sequencing was conducted at the International Livestock Research Institute (ILRI, Kenya) using R9.4.1 flow cells on an ONT MinION Mk1B sequencer. High-accuracy base calling was performed using Guppy basecaller^16^ (v4.1.1) generating 7.7 million reads totalling 37.5 Gbp of sequence that represents 52.4x of the estimated genome size from kmer analysis (Fig. 2).

#### Nanopore RNA sequencing

Two grams of young and disease-free leaves, stem and root tissues of AYB accession TSs11 were harvested and ground separately with mortar and pestle in liquid nitrogen. RNA was extracted separately from each sample using the RNA extraction protocol described by Xu *et al*.^17^ followed by removal of residual DNA using DNase I (RNase-free) kit (Thermo Fisher Scientific). The library was prepared following ONT SQK-PCS109 PCR-cDNA sequencing kit instructions. A total of 50 ng total RNA was transcribed using Maxima H Minus Reverse Transcriptase (Thermo Fisher Scientific). Full length transcripts were selected by PCR amplification using the LongAmp Taq Master Mix (NEB, UK), and the product was purified with AMPure XP beads (Beckman Coulter). Rapid Adapter was added to the amplified cDNA library. The libraries were sequenced at ILRI using R9.4.1 flowcells on the ONT MinION sequencers. Real-time data acquisition and high accuracy base-calling were conducted using the MinKNOW software with the Guppy basecaller (v5.0.2) generating 7.1 Gbp of sequence data from 17.1 million reads (Fig. 2).

#### Illumina DNA sequencing

We also generated Illumina short reads, mainly for kmer-based genome size estimation as described above, and for error correction of nanopore-based assembly described below. For this, seeds of AYB accession TSs11^10^ were germinated in a sealed petri dish containing filter paper moistened with tap water. The sprouted seedlings were transferred to soil and allowed to grow in the greenhouse facility at ILRI, Kenya, for a month. DNA was extracted from young disease-free leaves using a DNeasy Plant Mini Kit (Qiagen, Germany) following the manufacturer’s protocol, recovering a total of 10 µg. DNA was quantified using a Qubit 2.0 Fluorometer and dsDNA BR Assay (Invitrogen, UK), and integrity was confirmed by gel electrophoresis on a 0.8% agarose gel.

Three aliquots of 50 ng each of genomic DNA from the same plants were sheared and processed using the Nextera DNA Library Prep Kit (Illumina, USA) according to the manufacturer’s instructions. Three runs of paired-end (2 × 150 bp) sequencing on DNA from the same plant were performed on an Illumina MiSeq (Illumina) at ILRI, Kenya, to generate 25 Gbp of raw data, representing ~34.9x of the estimated AYB genome based on kmer analysis.

### De novo assembly

Initial draft genome assembly was done using the ONT long reads generated. Briefly, the reads from 18 ONT runs were concatenated and seqkit^18^ rmdup used to check for any duplicates (for runs that were basecalled twice). De-duplicated reads were passed as input to Flye *de novo* long read assembler (v2.9)^19^ with default parameters, generating 10,329 contigs with total assembly length of 701.6 Mbp (Fig. 2). For error correction, the draft assembly was polished with Illumina short reads generated from the same AYB accession (TSs11). This was performed using HyPo hybrid polisher^20^ (v1.0.3) with parameters -s 700 m -c 30 -p 96 and -t 64. This computational work was performed at ILRI, Kenya. The polished draft assembly had an N50 of 781,337 bp, and a total assembly length of 701.3 Mbp (Table 1) which represents 98% of the estimated genome size from kmer analysis. The draft assembly was further scaffolded using chromatin conformation capture (HiC) data as described below.

**Table 1:** AYB assembly statistics before and after HiC scaffolding.

Assembly Metric	Polished Assembly
Polished Assembly	
Number of contigs (>500 bp)	10,329
Total assembly length (bp)	701,349,621
N50 (bp)	781,088
L50(bp)	194
Longest contig(bp)	9,386,731
HiC Scaffolded assembly	
Number of scaffolds (>500 bp)	11 chromosome-scale, plus 8422 shorter scaffolds/contigs
Total length of top 11 scaffolds (bp)	649,801,261
N50 (bp)	69,519,929
L50(bp)	4
Longest scaffold (bp)	107,191,003
Mapping rate of genomic ONT reads	98.91%

### Hi-C scaffolding

Hi-C scaffolding was performed by Phase Genomics (Seattle, USA) using the Proximo Hi-C 2.0 Kit. For this, 1 g of fresh leaves from young AYB accession TSs11 plants were frozen in liquid nitrogen, ground to powder and cross-linked using 1% formaldehyde solution before being sent to Phase Genomics for library preparation following the manufacturer’s protocol. Sequencing of the Hi-C library was performed at Phase Genomics using Illumina HiSeq 4000 sequencer, generating a total of 275,166,448 paired-end reads. Reads were aligned against the polished assembly using BWA-MEM^21^ specifying the options -5SP and -t 8, and the other parameters set to the defaults. PCR duplicates were mapped using SAMBLASTER^22^ and were later excluded from the analysis. Non-primary and secondary alignments were flagged and filtered with Samtools^23^ using the -F 2304 filtering flag. Putative misjoined contigs were broken using Juicebox^24^ based on the Hi-C alignments, and the same alignment procedure was repeated from the beginning on the corrected assembly. Phase Genomics Proximo Hi-C genome scaffolding platform was used to create chromosome-scale scaffolds from the corrected assembly following the method similar to that described by Bickhart *et al*.^25^. Ordering of the scaffolds into pseudomolecules was done by LACHESIS^26^.

The scaffolded assembly contains 11 pseudomolecules (corresponding to the 11 AYB chromosomes) with 649.8 Mbp of sequence, and N50 of 69.5 Mbp (Table 1). An additional 8,422 short contigs with total and average length of 51.8 Mbp and 6.1 Kbp, could not be anchored into chromosomes. Summary statistics and evaluation of the completeness of the chromosome-scale genome assembly was performed using QUality ASsessment Tool (QUAST)^27^ (ver 5.0.2) and Benchmarking Universal Single-Copy Orthologs (BUSCO)^28^ (v5.2.2), respectively (see Technical Validation section).

Chloroplast and mitochondrial sequences were extracted from the raw illumina reads using GetOrganelle (v1.7.4.1) with default options^29^. The resulting contigs were used as queries for BLAST search against the final assembly with a percentage identify threshold of 70%. A subset of 41 of the 8422 unanchored contigs had >99% alignment coverage to contigs derived from GetOrganelle and were considered as organelle contigs. We also found organelle sequences of up to 9 kb integrated into the assembled pseudomolecules. Transfer of organellar DNA to the nuclear genome actively occurs in plants^30^. The alignment of Illumina reads and the lack of assembly gaps across the junctions of these insertions suggest that these sequences are likely true organellar integrants in the nuclear genome.

### Synteny with genomes of closely related plant species

**Fig. 4:**
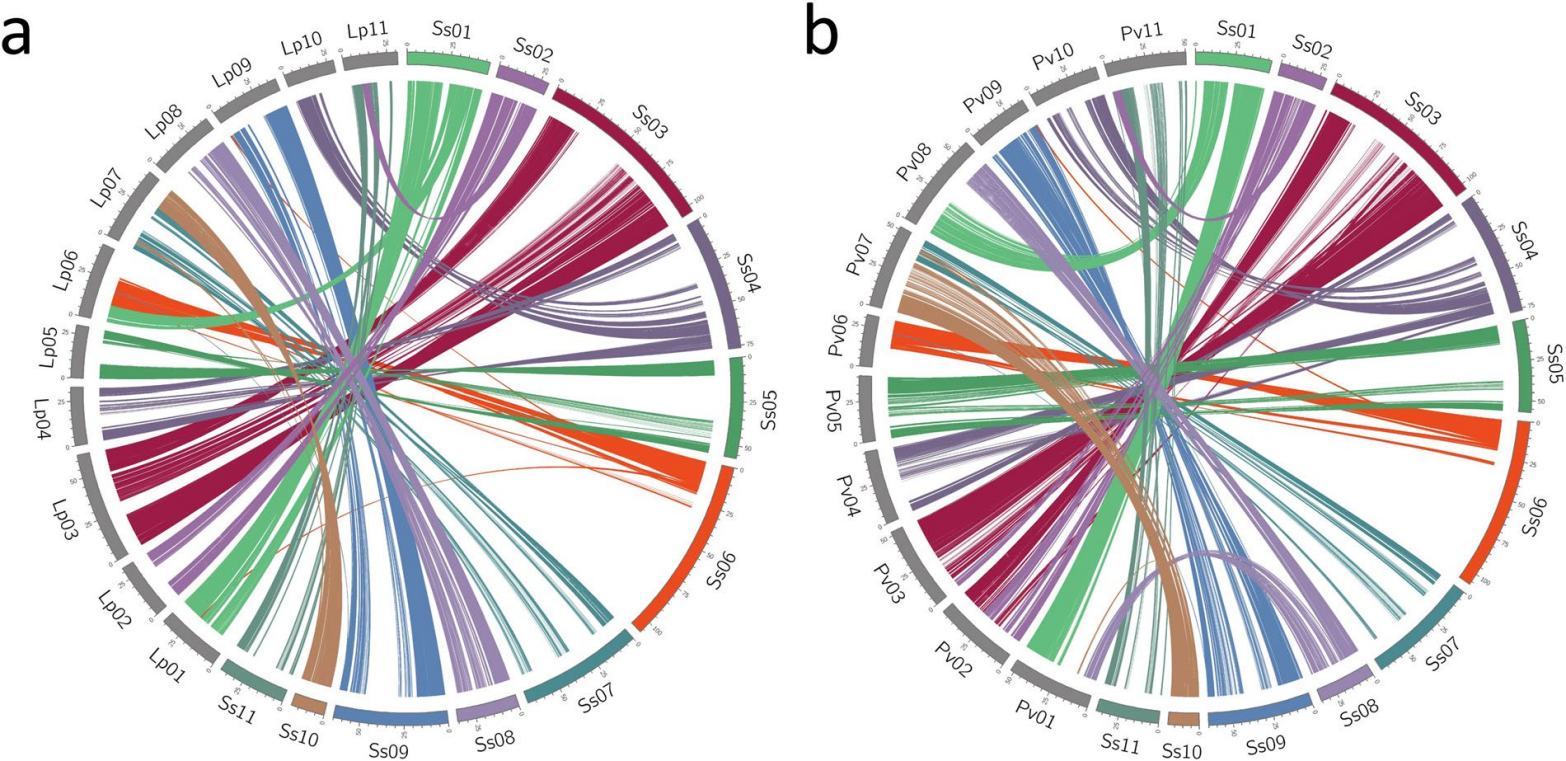
AYB synteny to related legumes. Syntenic relationships between AYB chromosomes to the genomes of (**a**) lablab (*Lablab purpureus*) and (**b**) common bean (*Phaseolus vulgaris*). Where possible, AYB chromosomes were renamed to reflect these syntenic relationships.

We examined the syntenic relationship between the HiC-scaffolded genome of AYB and the genomes of closely related species, including common bean (*Phaseolus vulgaris)*^31^ and lablab (*Lablab purpureus)*^32^. For this, long-read based genome assemblies and annotation datasets for *P. vulgaris* (PhaVulg1_0) and *L. purpureus*^32^ were obtained from Ensembl Plants^33^ and e!DAL^34^, respectively. The AYB protein dataset was compared to the common bean and lablab protein datasets using blastp from the BLAST v2.7.1 package with the parameters: -max_target_seqs. 1 -evalue 1e-10 -qcov_hsp_perc 70. The MCScanX^35^ algorithm was subsequently used to identify collinear blocks between the AYB-phaseolus and AYB-lablab genome pairs, specifying the parameters -s 20 and -m 10. Visualisation of synteny linkages was made with circos^36^ (v0.69-4). Six of the AYB chromosomes show direct one-to-one syntenic relationships with lablab and common bean chromosomes, while the other five AYB chromosomes show syntenic relationships with two or more chromosomes of the lablab or common bean genomes (Fig. 4). Based on these syntenic relationships, we assigned chromosome names to the Hi-C-scaffolded AYB pseudomolecules.

### Repeat annotation

**Fig. 5:**
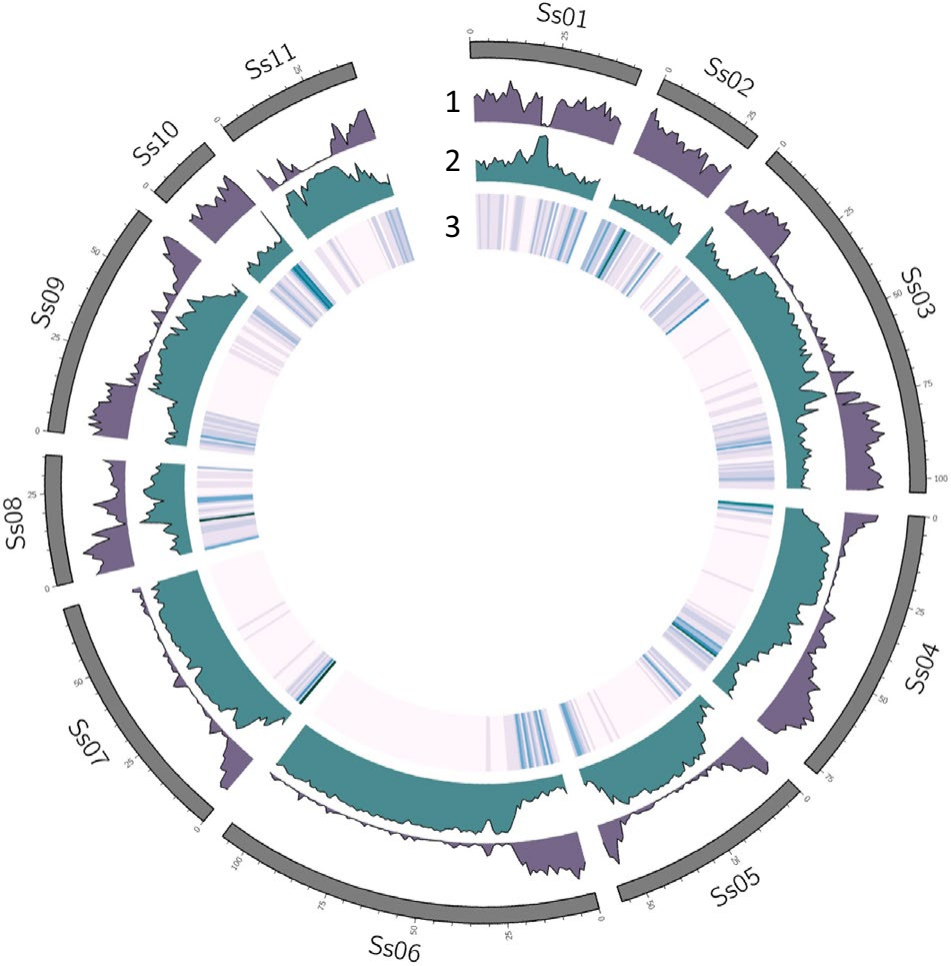
Gene, repeat and marker distribution in the AYB genome. The outer to the inner tracks show gene density, repeat density, and heatmap of marker distribution.

**Fig. 6:**
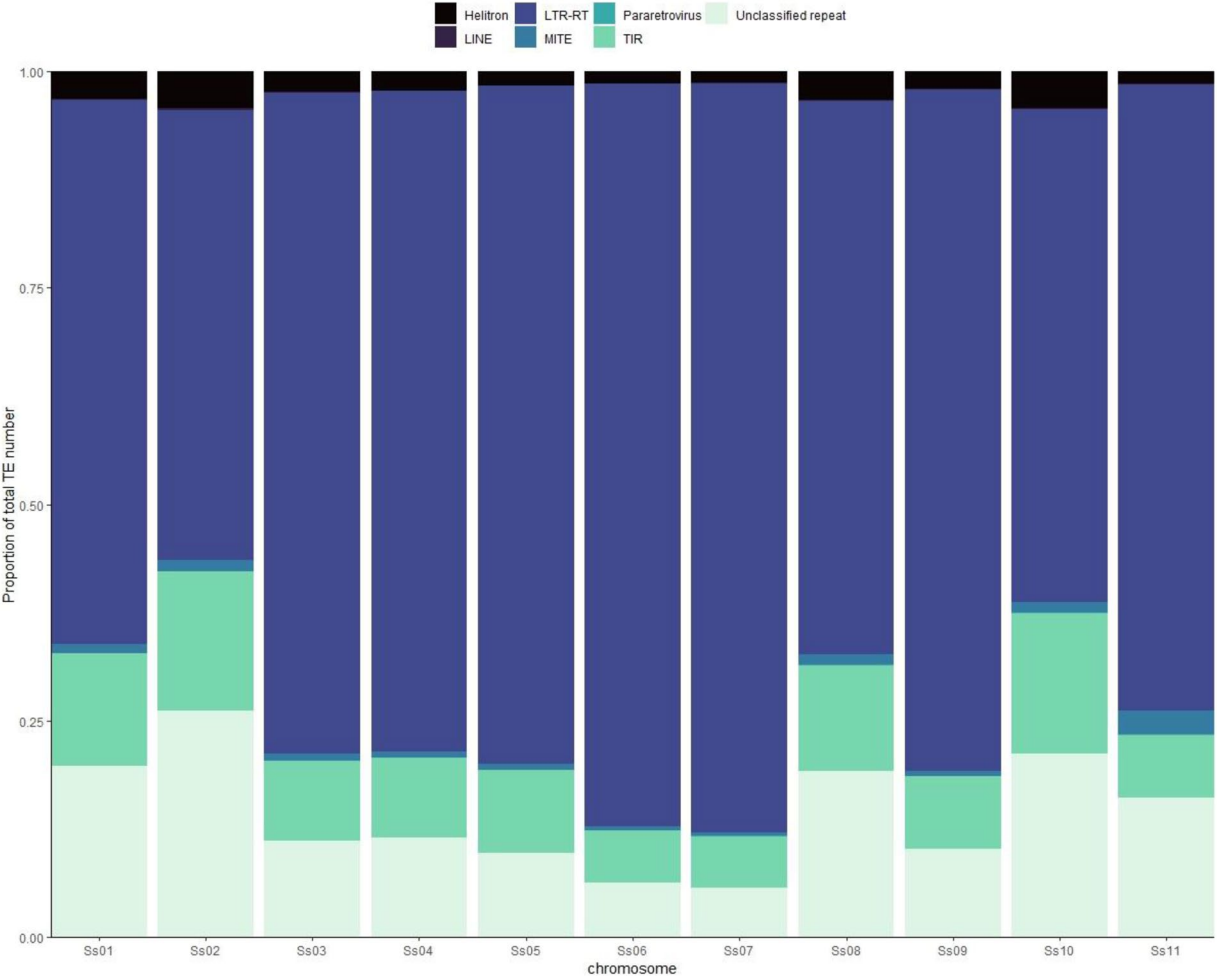
Distribution of transposable elements across the AYB genome. Chromosomal repeats content in the AYB genome showing proportional abundance of identified transposable element Orders on each chromosome.

The Extensive De novo TE Annotator^37^ (EDTA v1.9.7) pipeline was used to annotate transposable elements (TE) in the AYB genome. The pipeline incorporates different tools to annotate TE classes found in plant genomes using structure and homology-based detection methods. The tools include LTRharvest^38^, LTR_FINDER^39^, LTR_retriever^40^, TIR-Learner^41^, HelitronScanner^42^, RepeatModeler2^43^ and RepeatMasker^44^. The outputs of each tool are combined and filtered into a comprehensive non-redundant TE library. The inbuilt genome annotation function in EDTA was then used to produce a final non-overlapping repeat annotation file for the AYB genome. Data visualisation and summary were carried out using the Tidyverse suite^45^. In total, 624,517 TEs and 78,100 unclassified repeats accounting for 74.08% of the total assembly were identified across the genome (Table 2, Figs. 5, 6). We also used Tandem Repeat Finder^46^ to search for tandem repeats in the AYB genome. We identified 178,561 short sequence repeats (SSR) with greater than 90% match filter, comprising 53,524 microsatellites, 113,701 minisatellites and 11,336 satellites. Some of the unclassified repeats from EDTA (20,465 repeat, 26.2%) overlap with these SSR suggesting that they are tandem repeats.

**Table 2:** The number of TEs, TE families and the proportion of occupied assembly length by different classes of repeats identified and annotated in AYB.

Class	Order	Superfamily	Number of TEs	Number of Families	% of Assembly
**Class I**	LTR-RT	Copia	172471	3149	22.01
		Gypsy	183229	1488	24.13
		unknown	187360	1379	17.73
	LINE	unknown	351	6	0.04
**Class II**	TIR	CACTA	28514	494	2.4
		MUDR-Mutator	20256	264	1.23
		PIF-Harbinger	800	23	0.08
		Tc1-Mariner	675	25	0.03
		hAT	10466	116	0.59
	MITE	CACTA	39	32	0
		MUDR-Mutator	1255	174	0.03
		PIF-Harbinger	45	3	0
		Tc1-Mariner	2	2	0
		hAT	4496	22	0.21
	Helitron	Helitron	14242	27	1.44
**Other**	Pararetrovirus		316	1	0.01
	Unclassified repeat		78100	702	4.15
**Total**			**702617**	**7907**	**74.08**

### Gene prediction and functional annotation of genome

We combined transcript and protein homology evidence to annotate the gene content of the AYB genome. The transcript evidence was generated from 17,117,377 ONT RNA reads totalling 7.1 Gbp of sequencing data, used for *de novo* assembly of 60,249 transcripts. Briefly, Minimap2^47^ (v2.22) was used to index the AYB genome assembly and the RNA reads were mapped to the indexed assembly with parameters “*-ax splice -uf -k14*” that allows for splice-aware alignment. Samtools^23^ (v1.9) was used to sort mapped reads by coordinates, which were used to assemble transcripts using Stringtie2^48^ (v2.0.1). Transdecoder^49^ (v2.0.1) was then used to identify candidate CDS regions and select transcripts with a minimum protein length of 100 amino acids.

To identify protein coding genes, we provided the de novo assembled transcripts and protein datasets from four well-annotated plant genomes (*Arabidopsis thaliana* TAIR10, *Phaseolus vulgaris* v1.0, *Glycine max* v2.1, *Vigna_angularis* v1.1, all downloaded from Ensembl plants release-53^33^) together with the soft-masked (for repeats) AYB genome as inputs to Funannotate^50^ (v1.8.11). We used Funannotate ‘*predict’* with parameters “*--repeats2evm, --max_intronlen10000, --busco_db embryophyta, --optimize_augustus*”. Funannotate *‘predict’* uses *ab initio* gene predictors Augustus^51^, PASA^52^, SNAP^53^ and GlimmerHMM^54^ together with protein sequences as evidence to predict genes. Gene predictions from all four *ab initio* predictors are passed to EVidenceModeler^55^ with various weights for integration. This resulted in 30,840 coding gene models, comprising 31,614 transcripts, with a median exon length of 231 bp and a median of three exons per transcript (Table 3). Additionally, we detected 774 non-overlapping tRNA-encoding genes using tRNAscan-SE^56^ for tRNA prediction. The gene and transposable element distribution across the genome are inversely correlated (Fig. 5).

**Table 3:** Statistics of genes and transcripts predicted for AYB using the Funannotate pipeline.

Statistic	Number
Number of gene models	30,840
Number of transcripts	31,614
Mean transcripts per gene	1
Number of exons	158,862
Mean exons per mRNA	5.13
Mean exon length	218.84
Mean CDS length	1,121.77
Mean intron length	580.37
Number of genes with functional annotations	25,241

Protein domains were annotated using InterProScan-5.25-64.0^57^ based on InterPro protein databases, including TIGRFAM, SUPERFAMILY, PANTHER, Pfam, PRINTS and ProDom. We also used eggNOG-mapper^58^ (v2.1.7) to annotate predicted gene models. Funannotate *‘annotate’* uses results from InterProScan and eggNOG-mapper to annotate putative functions of protein sequences using PFAM^59^, UniProtKB^60^ and Gene Ontologies^61^ databases. In total, functional descriptions were assigned to 25,241 (81.85%) of the genes.

## Data Records

The raw reads and genome assembly generated in this study are available from the European Nucleotide Archive (ENA) and NCBI databases under Bioproject accession: PRJEB57813. The Bioproject contains raw reads from the Illumina DNA sequencing experiment (ERX11864111^62^, ERX11864112^63^, ERX11864113^64^), Nanopore DNA sequencing experiment (ERX11749821^65^), Hi-C sequencing experiment (ERX12855778^66^) and Nanopore RNA sequencing experiment (ERX11824751^67^, ERX11824752^68^, and ERX11824835^69^). The accession number of the genome assembly is GCA_963425845^70^ with individual chromosome accessions ranging from OY731398 to OY731408. Files for the annotation (gff3), unscaffolded contigs, and organellar contigs are available on Zenodo^71^ (10.5281/zenodo.13853757). The anchored GBS markers information is available on figshare^72^ (10.6084/m9.figshare.25118711.v1).

## Technical Validation

### Genome and annotation completeness

**Fig. 7:**
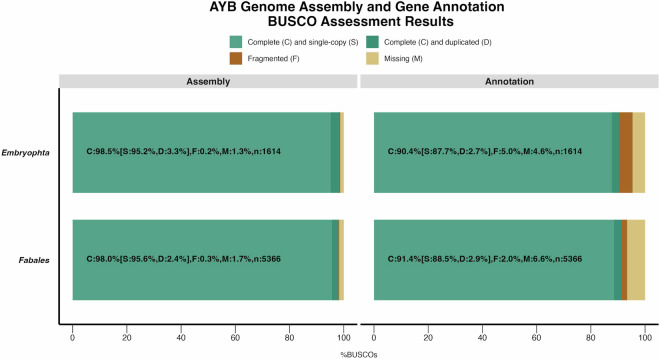
AYB Genome assembly completeness: BUSCO scores of the AYB genome and gene annotation using the embryophyta and fabales reference lineages.

We used Qualimap^73^ (v.2.2.2) to assess the quality of the assembly using read alignment of ONT long-reads to the soft-masked genome assembly generated with Minimap2^47^ (v2.22) using parameters “-ax map-ont”. A total of 98.91% reads were mapped (Table 1) with an average read coverage of 53.5x. This tallies with the pre-assembly coverage of 52.4x that we estimated for the ONT reads based kmer-based genome size estimate. We also evaluated the completeness of the genome assembly and annotation using BUSCO^28^ (v5.2.2). A highly conserved set of single-copy orthologs from embryophta_odb10 and fabales lineages were used as references. For the genome assembly, we obtained complete matches to 98.0% and 98.5% of the conserved single-copy orthologs in the fabales_odb10 and embryophyta_odb10 lineages, respectively (Fig. 7). Similarly, 90.4% and 91.4% of the conserved single copy orthologs showed complete matches to the predicted gene set of AYB (Fig. 7). These high percentages suggest a high degree of accuracy and completeness of the genome assembly and gene annotation.

### Marker mapping

We also examined the usefulness of the AYB genome for the positionally anchoring markers for genetic analyses. Previous efforts to anchor AYB markers to the common bean genome only mapped 17% of the markers to unique syntenic positions, limiting the strength of the genome-wide association analysis (GWAS)^74^. Using the chromosome-scale assembly of AYB as reference, we could anchor 92% of the 5,142 DArTseq-SNPs markers to unique positions in the AYB genome. The distribution of the markers across the genome tallies with the gene distribution affirming the gene-centric nature of the DArTseq pipeline (Fig. 5). This highlights the high quality of the AYB genome and its usefulness for the precise mapping of SNP markers and genome-wide allele mining for agronomic, biotic, abiotic and nutrition value traits in future AYB crop breeding.

## Data Availability

No custom code was used during this study. Only open-source software were used for the analyses reported. The software versions and custom parameters used (if different from default) are indicated in the Methods.
